# Response to “The Role of Patient‐ and Drug‐Related Factors in Oral Minoxidil and Pericardial Effusion: Analyses of Data From the United States Food and Drug Administration Adverse Event Reporting System”

**DOI:** 10.1111/jocd.70423

**Published:** 2025-08-25

**Authors:** Devyn Zaminski, Michael Garshick, Jerry Shapiro, Kristen Lo Sicco, Daniel R. Mazori

**Affiliations:** ^1^ New York University Grossman School of Medicine New York New York USA; ^2^ Leon H. Charney Division of Cardiology New York University Grossman School of Medicine New York New York USA; ^3^ Ronald O. Perelman Department of Dermatology New York University Grossman School of Medicine New York New York USA

**Keywords:** alopecia, hypertension, minoxidil, pericardial effusion

1

We read with great interest, “The Role of Patient‐ and Drug‐Related Factors in Oral Minoxidil and Pericardial Effusion: Analyses of Data From the United States Food and Drug Administration Adverse Event Reporting System” by Gupta et al., which investigated the incidence rates and factors associated with pericardial effusions reported during topical and oral minoxidil use [1]. The authors found an incidence rate of 1.27% and a dose‐independent relationship for pericardial effusions reported during oral minoxidil use [1]. For patients taking oral minoxidil for alopecia, we hope to broaden this conversation by discussing the possibility of underreporting of pericardial effusions and the utility of a refined approach to risk stratification and patient monitoring.

In the current study, Gupta et al. found that pericardial effusions were significantly associated with patients taking oral minoxidil for hypertension, whereas only 6.7% of pericardial effusions were reported in patients taking oral minoxidil for alopecia [1]. Unlike oral minoxidil use in hypertension management [2, 3], there are no standardized guidelines for risk stratification and monitoring for low‐dose oral minoxidil use in alopecia. In the absence of obtaining baseline and serial volume status measurements (e.g., physical exams to assess for peripheral edema, B‐type natriuretic peptide levels) or echocardiograms, pericardial effusions during low‐dose oral minoxidil use for alopecia have been diagnosed after symptom development in two cases [4, 5]. In one study, patients taking oral minoxidil for alopecia were diagnosed with asymptomatic, trivial (only present during systole) or small (< 1 cm) pericardial effusions using a non‐diagnostic, point‐of‐care ultrasound at the same rate as control patients not taking oral minoxidil [6]. A few possible explanations for this finding exist: (1) the pericardial effusions were unrelated to oral minoxidil use, or (2) in the absence of baseline imaging for comparison, the pericardial effusions could have been related to oral minoxidil use, but were chronic and slow accumulating, which can prolong or evade diagnosis in the absence of routine cardiac imaging [6, 7]. In either case, the difference in monitoring strategies employed for different minoxidil indications is potentially contributing to underreporting of pericardial effusions when oral minoxidil is used for alopecia. Low‐dose oral minoxidil was previously shown to have minimal risk for systemic side effects such as pericardial effusions [8]; however, the dose‐independent relationship for pericardial effusions found by Gupta et al. raises an important question: should dermatologists adopt similar screening criteria and monitoring protocols for oral minoxidil use in patients with alopecia as those in place for resistant hypertension?

In resistant hypertension management, cardiologists and nephrologists have guidelines to direct oral minoxidil use. For example, when oral minoxidil is used for hypertension, the Food and Drug Administration and 2017 American College of Cardiology/American Heart Association guidelines recommend concomitant use of a loop diuretic and beta blocker to mitigate the risks of fluid retention and reflex tachycardia, respectively [2, 9]. In patients with chronic kidney disease being treated with dialysis, nephrology guidelines for oral minoxidil use recommend increased volume status monitoring [3]. A new Delphi consensus statement lists a history of tachycardia or other arrhythmia, hypotension, renal impairment, and current use of hemodialysis as precautions for low‐dose oral minoxidil use in alopecia [10]. In our experience, demographics like older age and/or medication regimens with multiple anti‐hypertensives are also risk factors. Despite this Delphi consensus statement, there is still a need for standardized guidelines for cardiovascular risk assessment and standardized protocols for cardiovascular monitoring in the setting of oral minoxidil use for alopecia. In patients with preexisting risk factors for pericardial effusions, collaboration between specialties (i.e., dermatology, primary care, cardiology, nephrology) may be beneficial and enable screening for comorbidities such as coronary artery disease or hypertension. Furthermore, incorporating elements from existing cardiology and nephrology guidelines may enhance care for those with preexisting risk factors or comorbidities. Possible considerations may include B‐type natriuretic peptide screening for volume status and baseline and/or serial echocardiograms.

As the off‐label use of oral minoxidil for alopecia increases, a collaborative approach between specialties to stratify risk and guide therapeutic monitoring is of growing utility. In the absence of standardized monitoring, the likelihood of underreporting of pericardial effusions during oral minoxidil use is increased, particularly if the risk is dose‐independent. Physicians should be cognizant of this possibility and may consider performing enhanced monitoring and closer consideration of comorbidities when prescribing oral minoxidil. Further research should focus on examining a potential dose‐related risk threshold for side effects, identifying susceptible patient populations, and determining the utility of baseline screening and routine cardiovascular monitoring for high‐risk patients taking oral minoxidil for alopecia. Established protocols and standardized guidelines for oral minoxidil use could help minimize adverse events, while maximizing the efficacy of this therapy for alopecia. We commend Gupta et al. for their important contribution to the literature.

## Author Contributions

All authors have seen, approved, and contributed significantly to this manuscript.

## Ethics Statement

The authors have nothing to report.

## Conflicts of Interest

Dr. Lo Sicco is a consultant for Pfizer and Aquis and is a board member of the Scarring Alopecia Foundation and the American Hair Research Society. Dr. Shapiro is a consultant for Aclaris Therapeutics, Incyte, and Replicel Life Sciences. Drs. Lo Sicco and Shapiro have been investigators for Regen Lab and are investigators for Pfizer. Dr. Garshick is a consultant for BMS, Argenx, Agepha, and Kiniksa. All other authors have no conflicts of interest to disclose.

## Linked Articles

This article is linked to https://doi.org/10.1111/jocd.16732.

## Data Availability

No new data were generated or analyzed in support of this research.
